# Mode Transition of Droplet Formation in a Semi-3D Flow-Focusing Microfluidic Droplet System †

**DOI:** 10.3390/mi9040139

**Published:** 2018-03-21

**Authors:** Yan Wu, Xiang Qian, Min Zhang, Ying Dong, Shuqing Sun, Xiaohao Wang

**Affiliations:** 1 Division of Advanced Manufacturing, Graduate School at Shenzhen, Tsinghua University, Shenzhen 518055, China; yan-wu15@mails.tsinghua.edu.cn (Y.W.); zhang.min@sz.tsinghua.edu.cn (M.Z.); dong.ying@sz.tsinghua,edu.cn (Y.D.); wang.xiaohao@sz.tsinghua.edu.cn (X.W.); 2 Institute of Optical Imaging and Sensing, Shenzhen Key Laboratory for Minimal Invasive Medical Technologies, Graduate School at Shenzhen, Tsinghua University, Shenzhen 518055, China; sun.shuqing@sz.tsinghua.edu.cn; 3 The State Key Laboratory of Precision Measurement Technology and Instruments, Tsinghua University, Beijing 100084, China

**Keywords:** droplet, flow-focusing, surfactant concentration, uniformity, pressure ratio

## Abstract

Uniform droplets have significant potential in many biological applications due to their higher surface area to volume ratio. This paper proposed a semi-three-dimensional (sime-3D) flow-focusing microfluidic system, which was fabricated using the multi-layer soft lithography method. Based on the semi-3D structure, we focus on droplets formation modes and droplet uniformity at different bulk concentration of surfactant. The relationships between droplets uniformity, droplets breakup processes (jetting mode, dripping mode and tip-streaming mode) and surfactant concentration was investigated. It was found that three droplet generation modes occur through adjusting the pressure ratio in two inlet channels and the concentration of surfactant in continuous phase liquid. The jetting mode would transform to the dripping mode or the tip-streaming mode as the pressure ratio in different surfactant concentrations increased. Furthermore, the uniformity of droplets could be improved through the transition of jetting to dripping mode. We assumed that the uniformity declined through the transition of jetting to tip-streaming, and explored the specific transitions from jetting to dripping mode and tip-streaming mode. Dripping mode leads to high droplet uniformity, and generation frequency decreases with increasing pressure ratio. Tip-streaming mode is considered as an extreme state of jetting mode, leading to higher formation frequency and smaller droplet size at low uniformity.

## 1. Introduction

Due to the specific physical-chemical properties of micro- and nano-droplets, such as high surface-volume ratio and minute size, the microfluidic droplet technology is one of the fastest growing technological sectors and plays important roles in biological applications, such as drug and gene discovery [1], determining DNA structure [2], synthesis of biomolecules [3,4], and diagnostic testing [5]. High surface-volume ratio can cause a significant increase in reaction rate and the molecule’s reactivity; high uniformity reduces the experimental errors and ensures stability and quality. Therefore, minute size, high productivity and uniformity of droplets are the key parameters which researchers want to acquire most.

As a typical microfluidic method, flow-focusing technology offers a controllable environment for droplets production [6,7,8,9]. In the typical two dimensional (2D) flow-focusing chip, the dispersed phase flow is sheared from the lateral. In 2D systems, the formation of stable tip-streaming mode generally needs the support of high voltage electric field [8,10,11], or more precision for pressure control [12]. A semi-3D flow-focusing microfluidic system was proposed by Jeong and co-works to generate uniform and monodispersed size-controllable poly (ethylene glycol) diacrylate (PEGDA) droplets via different breakup process [12]. Compared with 2D systems, the semi-3D chip provides shallow dispersed phase channel and an orifice, deep continuous phase channel and downstream channel, so that the dispersed phase flow is sheared form all directions. The semi-3D structure has the special advantage of being able to generate droplets from a nanometer to micrometer diameter range, because it allows a larger adjusting range for flow-focusing phase without the support of the electric field. However, PEGDA without dilution as the dispersed phase leads to an unstable tip-streaming mode [12]. Surfactant dissolved in the continuous phase liquid decreases the local surface tension, and plays a central role in the mode of droplet formation. In previous research, it was found that, when the bulk surfactant concentration is much higher than the critical micelle concentration, the interfacial tension is approximately invariable in the saturated state [13] and the tip-streaming mode can occur [14]. Dripping, jetting and tip-streaming modes could occur by changing the pressure ratio of two-phase flows in microchannel, and droplets were produced with a distribution from micro to sub-micro scale. However, in their work, the effects of key factors other than pressure ratio were not well defined. We observe heterogeneous droplets in figures of previous work [6,10,14].

In our work, we independently proposed a similar semi-3D flow-focusing microfluidic with slightly different structure for gas-liquid flow-focusing ionizing [15]. In this report, based on the same semi-3D structure with modifications, we focused on droplets formation at different bulk concentration of surfactant. We deeply investigated the relationship between droplets uniformity and generation frequency, droplets breakup processes (jetting mode, dripping mode and tip-streaming mode) and surfactant. According to the results of PEGDA droplet generation, when the pressure ratio is improved, the transition from jetting to dripping mode occurs when the bulk surfactant concentration is less than 10 vol %, and another transition from jetting to tip-streaming mode happens when the concentration is larger than 15 vol %; compared with jetting mode, dripping mode improves droplet uniformity, while tip-streaming mode reduce the uniformity, and a quantitative measurement for the variation of PEGDA droplet uniformity is illustrated. We further explore the transition from jetting to dripping mode using trimethylolpropane ethoxylate triacrylate (ETPTA) as the dispersed phase, and observe the tip-streaming mode using deionized water as the dispersed phase, respectively. Comparing and observing the relationships of droplet formation frequency, droplet size and discrete position under tip-streaming and jetting mode, we think the tip-streaming mode can be considered as an extreme case of jetting mode, and obtain a qualitative conclusion: ‘the tip-streaming mode decreases the droplet uniformity and dripping mode increases the droplet uniformity’, strengthening the previous conclusion. The paper will be applied to provide useful, efficient and convenient design strategies for droplets generation. In addition, the PEGDA or ETPTA particle can be synthesized through photo-polymerization process under UV exposure and provides more possibilities for biological applications.

## 2. Materials and Methods

### 2.1. Experimental Design

#### 2.1.1. Device Layout and Microfabrication

Microfluidic device is fabricated using typical multi-layer soft lithography techniques. Briefly, the silicon mold was fabricated using different lithography mask and transferred to the PDMS layer through cast molding. The first layer was patterned on a silicon wafer by coating 2–3 mL SU-8 2025 negative photoresist (Microchem Co., Naton, MA, USA). The photoresist was exposed through a first photomask (a chrome plate on glass, fabricated by Qingyi Precision Mask Making Co., Ltd., Shenzhen, China), which provideed the designs of an 80 μm wide channel for introducing the dispersed phase and a 50 μm wide orifice. After post-baking and cooling, we repeated the coating prosecco with SU-8 2100, a more viscous negative photoresist on the top of the first layer. The photoresist was exposed through a second photomask, providing the designs of the continuous phase channel and the exit channels. An alignment mark on the photomasks was designed to align the patterns of these two layers using an aligner. The SU-8 photoresist layers are developed by immersing in SU-8 developer (propylene glycol methyl ether acetate: PGMEA) until the features become clear on the wafer. After hard-baking, the first layer is about 25 μm tall and the second layer is 130 μm.

The top and bottom PDMS half-devices with mirrored structures are prepared using PDMS in different weight ratio of PDMS base monomer and its curing agent (Sylgard 184, Dow Corning, Midland, MI, USA), typical 10:1 for top layer and 8:1 for bottom layer. Both layers are cured at 80 ${}^{\circ }$C for 1 h. Two inlets and one outlet holes were drilled into the top layer using a 0.75 mm diameter punch. After cleaning and oxygen plasma treatment, the top and bottom layers were bonded together, and the features are aligned by viewing through a stereo microscope. The final bonded microfluidic chip was cured in an oven at 120 ${}^{\circ }$C for one day to enhance the strength.

#### 2.1.2. Microfluidic Emulsification

Both the dispersed phase and continuous phase solutions were supplied to the microchannels through a 2 mL reservoir or 200 μL Gel loading tip, pressured using a high precision microfluidic pressure controllers (MFCS, Fluigent, Paris, France) with a sensitivity of 0.35 mbar. The inlet holes were connected with a pressure controller using FEP tube and a short stainless steel tube. The droplets were produced by adjusting the pressure ratio of two phases. In general, we set the pressure of dispersed phase to a constant as the base-level pressure of the system, for example maintaining it at 50 mbar, then increaseed the pressure of the continuous phase until the emulsion breakup mode changed from the jetting to dripping or tip-streaming. All liquids were allowed to pass through for 10 min to stabilize and remove different droplets remaining within the outlet hole and tube.

#### 2.1.3. Characterization

An FEP tube was used to connect the outlet hole to the culture dish to collect the droplets. After finishing the collecting process, the sample was observed through the fluorescent microscope with 20X, 40X and 100X objects respectively. The digital fluorescent images captured by the high-speed camera were analyzed by ImageJ and Image Pro. The background of image was first subtracted based on the rolling ball method; Gaussian blur was applied to reduce the image noise; a black-white image can be acquired after adjustment of the thresholds and binary progress; watershed algorithm was used to separate the adhesion targets and the sphere objects in the image were extracted based on the edge detection method; finally, the diameter of each sphere object can be calculated. As a result, diameters and standard deviation of the diameters of sphere objects in each image can be estimated.

### 2.2. Materials

The dispersed phase liquid is pure hydrophilic poly (ethylene glycol) diacrylate (PEGDA, MW 255, Sigma-Aldrich, St. Louis, MO, USA), trimethylolpropane ethoxylate triacrylate (ETPTA, MW428, Aladdin, Shanghai, China) or deionized water containing Rhodamine B (95%, MW 479.01, Aladdin, Shanghai, China) at a concentration of 1 mmol·L${}^{-1}$ as the fluorescent tracer for visualization of the droplets. The continuous phase liquid is the mixture of Hexadecane (99%, Sigma-Aldrich, St. Louis, MO, USA) dissolving with different ratio of silicone-based nonionic surfactant (ABIL EM90, Degussa, Essen, Germany).

## 3. Results and Discussion

### 3.1. Semi-3D Flow-Focusing Microfluidic Device

**Figure 1:**
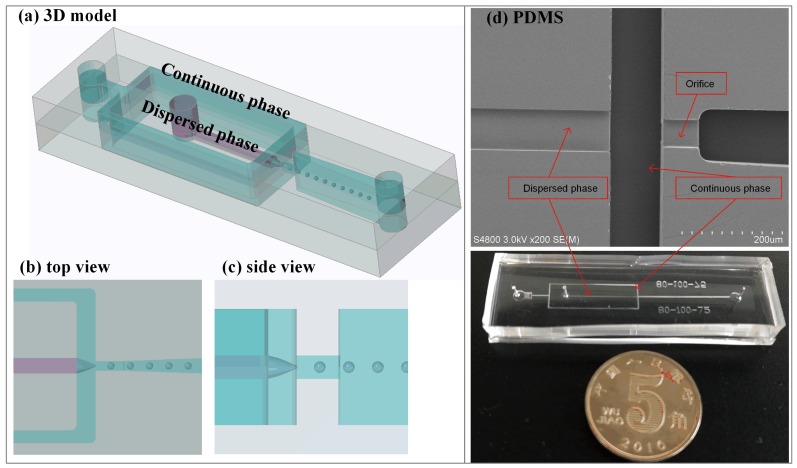
A semi-3D flow-focusing microfluidic device. (**a**) Schematic diagram of 3D model. (**b**,**c**) Top view and side view. (**d**) Structures of PDMS slab under SEM and monolithic microfluidic device.

**Figure 2:**
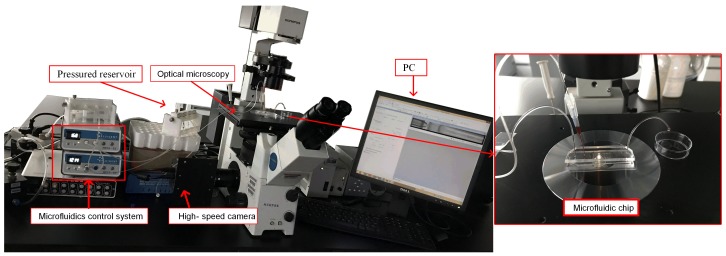
The configuration of experimental system.

The schematic diagram of the semi-3D flow-focusing microfluidic device is illustrated in Figure 1a–c. The practical monolithic device and structures of PDMS slab under SEM are shown in the Figure 1d. The channel height of the dispersed phase and the orifice is narrowed by 6 times compared to the height of the continuous phase and the downstream channel. As a result, the dispersed phase flow is sheared by the continuous phase liquid from all directions. In addition, the width is enlarged by 2 times compared to the orifice. The hydraulic resistance (R${}_{hyd}$) of the dispersed phase channel is larger than both the continuous phase channel and outlet channel (the orifice and downstream channel) in the design of the channels, while R${}_{hyd}$ of continuous phase channel is smallest. The R${}_{hyd}$ for the rectangular microchannel is calculated according to the following formula [16]:

(1) $$R_{hyd}=\frac{12\eta L}{wh^{3}\left(1-\frac{h}{w}\left(\frac{192}{\pi^{5}}\sum_{n=1,3,5}^{\infty }\frac{1}{n^{5}}\tan h\left(\frac{n\pi w}{2h}\right)\right)\right)}$$

where *L*, *w*, and *h* are the length, width and height of the channel, and η is the kinetic viscosity of the solution. In the semi-3D flow-focusing device, R${}_{hyd}$ of dispersed phase is $1.26\times 10^{14}$ Pa·s·m${}^{-3}$, and the sum of R${}_{hyd}$ of the orifice and downstream channel is $6.08\times 10^{12}$ Pa·s·m${}^{-3}$. The difference between the two R${}_{hyd}$ leads to a significant suppression for the retraction motions of the dispersed phase liquid cone, contributing to achieve the tip-streaming mode. The configuration of the experimental system is shown in Figure 2.

### 3.2. Droplets Formation

#### 3.2.1. Droplet Breakup Processes

**Figure 3:**
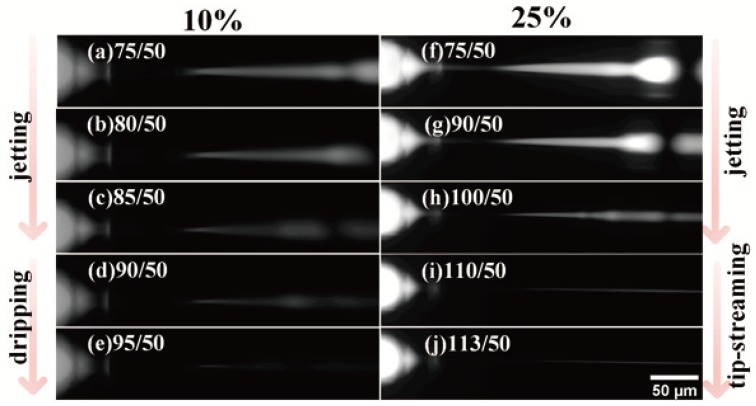
Representative fluorescent images of the transition of droplet breakup processes at bulk concentration of surfactant of 10 vol % and 25 vol %, including (**a**–**d**,**f**–**h**) jetting mode, (**e**) dripping mode and (**i**,**j**) tip-streaming mode.

Figure 3 shows the representative fluorescent images of the transition of droplet breakup processes, and the video of breakup process is shown in the Supplementary Materials (Video S1). The bulk concentration of surfactant changes from 5 vol % to 25 vol %, and the pressure ratio (the pressure of continuous phase (P${}_{C}$): the pressure of dispersed phase (P${}_{D}$)) changes from 1.4 to the ratio that makes the dispersed phase flow break off. For our experimental system, the experimental result shows that the PEGDA droplet is generated in the jetting mode at the beginning, whatever the bulk concentration is. The dispersed phase flow extends beyond the orifice and resembles a long jet. When the bulk concentration of surfactant is 5 vol % and 10 vol %, the emulsion breakup mode will change from jetting to dripping mode. The dispersed phase liquid cone breaks into discrete droplets at the cone tip. However, when the bulk concentration of surfactant exceeds 15 vol % (including 15 vol %, 20 vol % and 25 vol %), the transition from jetting to tip-streaming mode occurs. A very thin liquid thread forms from the end of the stable conical tip, and the discrete droplets cannot be observed at a frame rate of 690. Five pressure ratios of transition are recorded in the Table 1.

#### 3.2.2. Uniformity Based on Pressure Ratio and Surfactant

**Figure 4:**
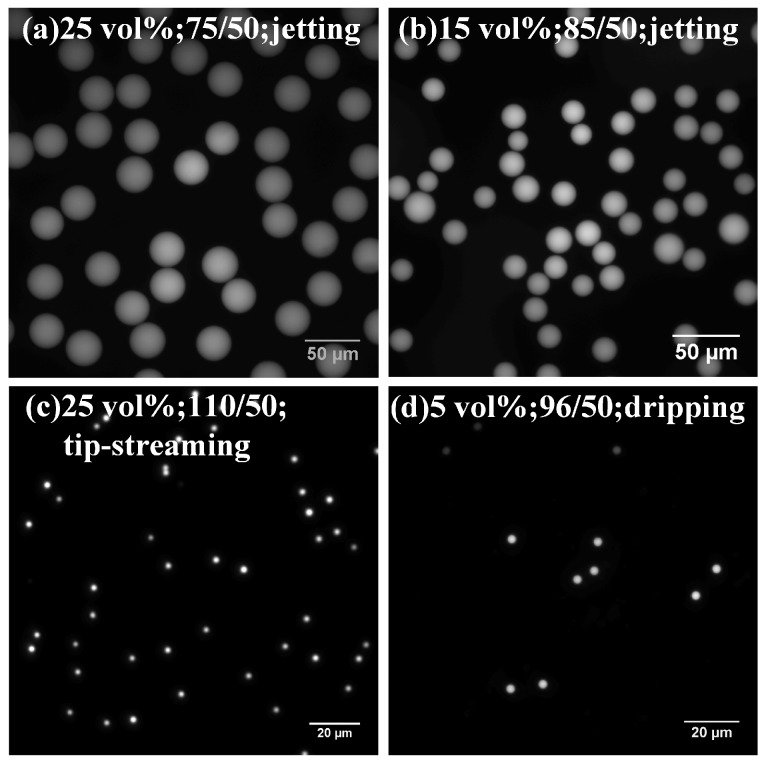
Fluorescent images of PEGDA droplets produced under different conditions. The average diameters and coefficients of variation of droplets in (**a**–**d**) are 32.15 μm and 4.44%, 17.50 μm and 9.62%, 3.29 μm and 16.15%, 3.36 μm and 5.34%.

PEGDA droplets in different size and uniformity can be produced through a semi-3D flow-focusing device adjusting the pressure ratio and the surfactant concentration. The PEGDA droplets size becomes small with increasing pressure ratio, as shown in many previous works [9,12,17]. The diameter range of the PEGDA droplet varies approximately from 1 μm to 40 μm. Figure 4 illustrates the fluctuations of droplet size and uniformity at that three breakup processes with different bulk surfactant concentration.

**Figure 5:**
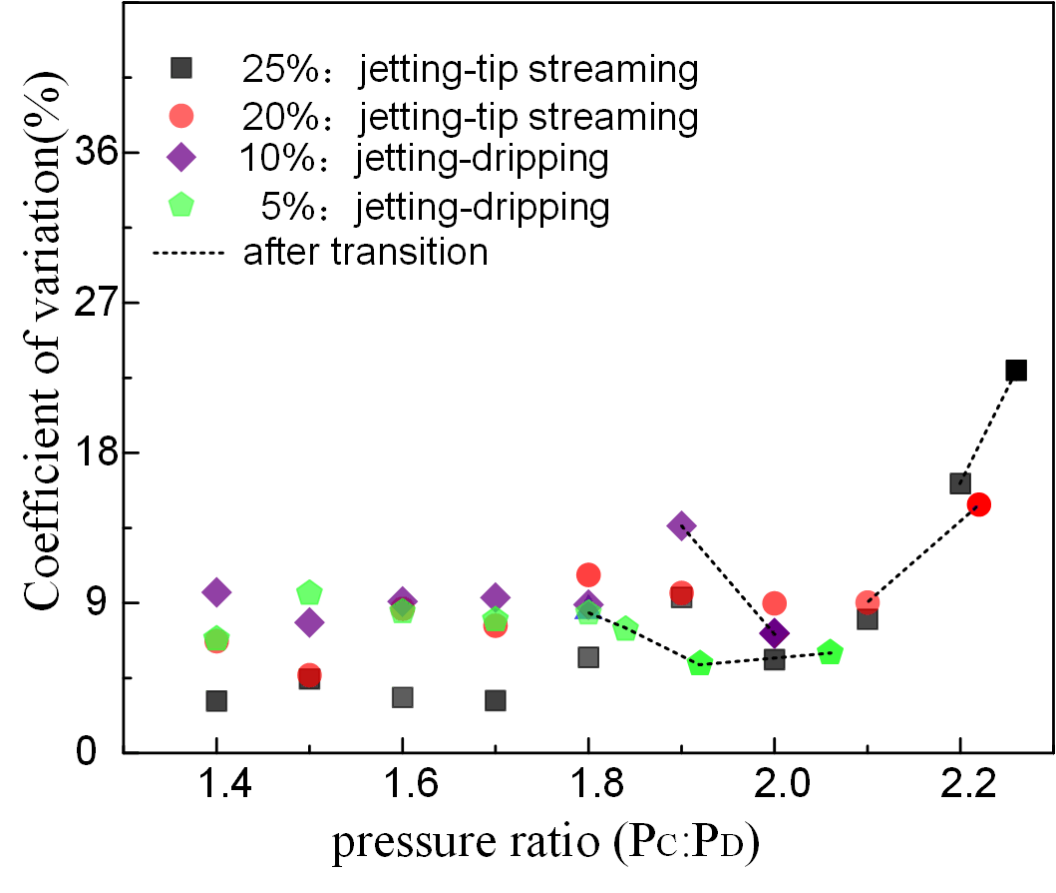
The coefficients of variation for poly (ethylene glycol) diacrylate (PEGDA) droplets on the basis of the pressure ratio and the bulk concentration of surfactant. CV = standard deviation/mean.

The coefficients of variation (CV) is the ratio of standard deviation to mean, reflecting the droplet uniformity without impact of scale. The value of CV is inversely proportional to the uniformity. Figure 5 illustrates the fluctuation of droplets uniformity at the emulsion breakup process with different bulk surfactant concentration. The increasing pressure ratio promotes the fierceness of emulsion process, leading to declining uniformity. The bulk surfactant concentration has no significant effect on the CV for PEGDA droplets before the jetting mode transforms to another mode. However, the coefficient of variation of PEGDA droplets becomes lager after the jetting mode changes to the tip-streaming mode, while the CV decreases if the emulsion process changes tothe dripping mode in low bulk surfactant concentration.

Below we explore the effect of surfactant and pressure ratio to verify the above conclusion, because it is hard to characterize the CV of smaller droplet. A few micron PEGDA droplets are observed through the fluorescent microscope with 40X and 100X objects, so there are only approximately twenty pixels in radius. In addition, the formation frequency (only dripping mode) and the volume fraction of the droplet are low, so that the sample size of a smaller droplet is less. Hence, the accuracy of a smaller droplet CV is lower than dozens of micron droplets. We focus on the droplet formation in jetting mode and dripping mode, which are caught easily under the present microscope. Then, we will determine the relationship of these three modes by analyzing the formation frequency, droplet size and discrete position.

### 3.3. Transition from Jetting to Dripping Mode

**Figure 6:**
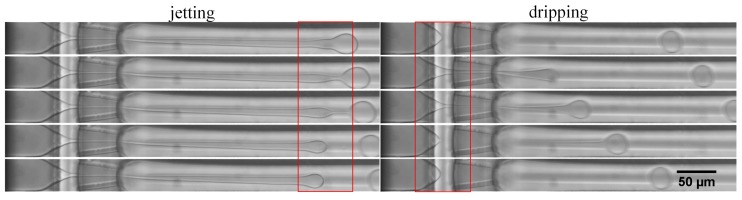
The trimethylolpropane ethoxylate triacrylate (ETPTA) droplet formation process through jetting and dripping mode. The bulk concentration of surfactant is 5 vol %.

PEGDA is replaced by ETPTA as the dispersed phase liquid, because there is only one transition of droplet breakup processes (jetting to dripping), whatever the bulk concentration is (below 26 vol %). Therefore, the effects of surfactant and pressure can be defined accurately. Figure 6 shows the ETPTA droplet formation process through jetting and dripping mode. Under jetting mode, the dispersed phase flow extends beyond the orifice and forms a long laminar jet, then the droplet forms gradually and breaks away from the jet tip. Unlike the jetting mode, the discrete position is the cone tip before the orifice under the dripping mode. The droplet is squeezed by the orifice and forms ‘a little tail’, resembling a tadpole.

**Figure 7:**
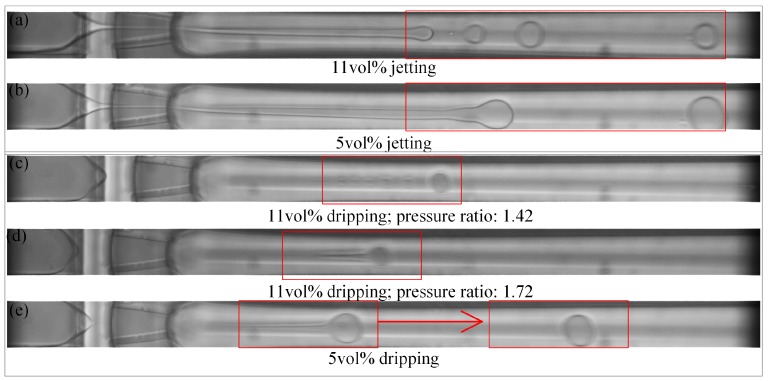
The droplet formation under jetting and dripping mode for different bulk concentration of surfactant: 11 vol % and 5 vol %.

Different bulk concentrations of the surfactant lead to differencea in droplet formation, both under jetting and dripping mode. The droplet uniformity clearly becomes worse with increasing surfactant concentration, which is illustrated in Figure 7. When the ETPTA droplet is generated in the jetting mode, the jet tip is more volatile and breaks into smaller droplets of varying sizes for 11 vol % (Figure 7a). When the ETPTA droplet is generated in the dripping mode, the ‘little tail’ of ‘tadpole’ will dissociate droplets in different sizes for 11 vol % (Figure 7c), but, the discrete situation does not occur under faster flow when the pressure ratio increases (Figure 7d). However, the ‘tail’ will gradually merge with the main droplet and there will generate only one droplet at 5 vol % (Figure 7e), as seen in Figure 7d. Videos S2 and S3 in the Supplementary Materials display that the ETPTA droplet generates for 5 vol % and 11 vol % under different pressure ratio.

**Figure 8:**
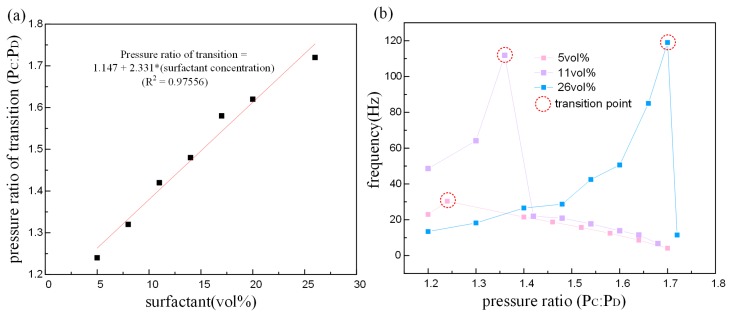
(**a**) The corresponding pressure ratio of transition from jetting to dripping mode on the basis of the surfactant concentration. (**b**) ETPTA droplet generation frequency.

The pressure ratios for transition from jetting to dripping mode under different surfactant concentration are shown in Figure 8a. The relationship between pressure ratio of transition and surfactant concentration is estimated with a linear fit. Higher surfactant concentration leads to higher pressure ratio for transformation. In other words, the dripping mode happens more easily with lower surfactant concentration. Furthermore, the differences in the formation frequency also reflect the transition from jetting to dripping. Image analysis reveals the droplet formation frequency for different pressure ratio and surfactant concentration, which are plotted as a function of the pressure ratio in Figure 8b. It can be observed that the generation frequency increases as the pressure ratio increases under jetting mode. When the jetting mode transforms to dripping, frequency declines sharply and immediately (excepting 5 vol %), then gradually decreases with increasing pressure ratio.

**Figure 9:**
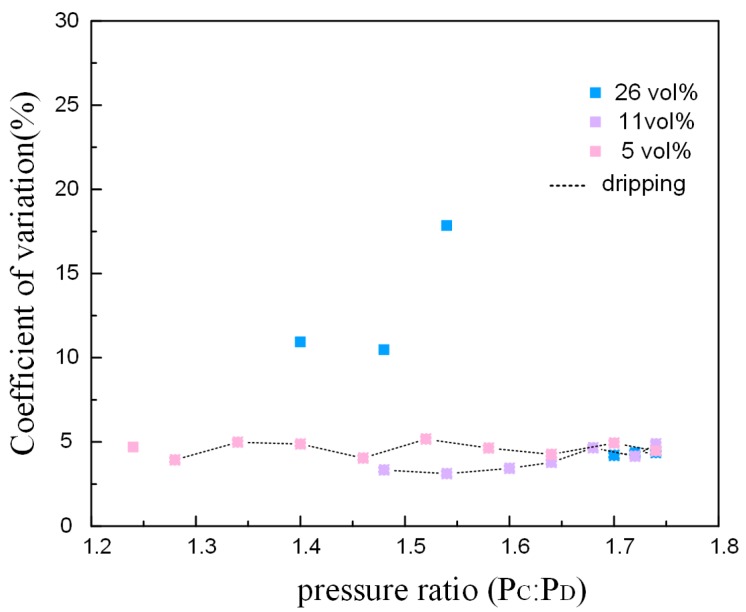
The CV of ETPTA droplets on the basis of pressure ratio and the bulk concentration of surfactant. CV = standard deviation/mean.

**Figure 10:**
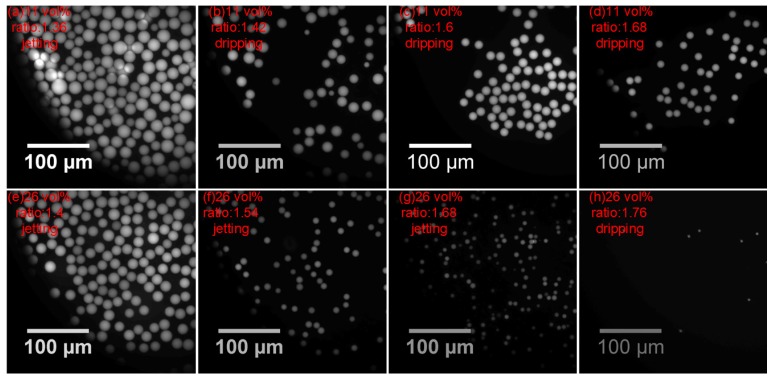
Droplet generate under different states. The CVs of (**b**) and (**g**) are difficult to estimate.

The droplet CV is plotted as a function of the pressure ratio, showing in Figure 9. The surfactant has more influence than the pressure ratio in the droplet uniformity. The CV of ETPTA droplet is higher under the jetting mode. In other words, the uniformity is lower under the jetting mode than the dripping mode. However, the pressure ratio and surfactant concentration both have no significant impact on the CV under dripping mode. The uniformity is lower under the jetting mode than the dripping mode. When the pressure ratio is close to the pressure ratio of transition, the more volatile dispersion of the jet causes lower droplet uniformity (Figure 10g), and the dispersion of ‘tail’ also bring on higher CV under dripping mode (Figure 10b). When the flow rate increases as the pressure ratio increases, the discrete state of ‘tail’ is improved, leading to higher droplet uniformity (Figure 10c,d,h).

### 3.4. Discussion

In our semi-3D flow-focusing microfluidic system, undiluted PEGDA solution is used as the dispersed phase liquid, increasing the difficulty to form a stable droplet breakup process. Increasing the concentration of surfactant leads to the more stable tip-streaming mode and an decrease in the droplet size. Three breakup modes will occur by adjusting pressure ratio and surfactant concentration. The interface becomes complicated when there is a large viscosity contrast between these two phases’ flows [18]. Comparing the droplet generation processes under the jetting mode, the droplet uniformity clearly worsens with increasing surfactant concentration. In other words, the instability becomes greater with increasing surfactant concentration. Then we find that more uniform droplets are generated under the dripping mode. The same conclusion can be obtained by exploring the transition from jetting to dripping mode, using ETPTA as the dispersed phase liquid. When the surfactant concentration is below 5 vol %, both the pressure ratio and droplet formation mode have no great effect on the droplet uniformity and generation frequency. However, increasing the surfactant concentration makes the transition from jetting to dripping mode more difficult, and the droplet uniformity is lower than the uniformity at 5 vol % under the jetting mode.

**Figure 11:**
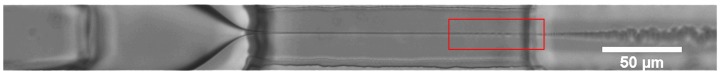
Tip-streaming mode.

To get a better observation for tip-streaming process, deionized water is used as the dispersed phase liquid and a longer orifice is designed, because deionized water and hexadecane can be form a clear interface of liquid-liquid, and deionized water significantly reduces the difficulty to form the stable tip-streaming mode. Figure 11 and Video S4 in the Supplementary Materials show the tip-streaming mode. It is worth noting that fast dripping occurs at the end of the orifice, between dispersion positions of the jetting mode and dripping mode. Smaller droplets are generated at a high production rate.

Under the jetting process, high pressure ratio increases the generation frequency and reduces the droplet size. Taking into account higher droplet formation frequency, smaller droplet size and the discrete position under tip-streaming, we think the tip-streaming mode can be considered as an extreme case of the jetting mode. Therefore, although the accuracy of previous quantitative measurements for the PEGDA droplets is low, it can be convinced that tip-streaming mode will decrease the droplet uniformity. However, under tip-streaming mode, droplet size is at a nanoscale so that the low uniformity has no significant influence on the later application stage.

As pure PEGDA is the dispersed phase liquid, high concentration of surfactant contributes to tip-streaming mode formation. Both dripping mode and tip-streaming mode can produce submicron PEGDA droplets. However, the submicron droplets are generated at a high frequency and low uniformity under tip-streaming mode, while the opposite result occurs under dipping mode. For our semi-3D flow-focusing device, generation of PEGDA droplets with better uniformity at a high frequency is a challenge to be solved.

## 4. Conclusions

In our semi-3D flow-focusing microfluidic geometry, the submicron PEGDA droplet is produced in the tip-streaming mode and dripping mode. The bulk concentration of surfactant is the key parameter for mode transition when the pressure ratio of two-phase flows in microchannel increases. The jetting mode changes to dripping mode at low concentration, while tip-streaming mode occurs at high concentration. The dripping mode leads to high droplet uniformity, and generation frequency decreases with increasing pressure ratio. However, when the surfactant concentration increases, jetting mode will lead to low uniformity, and generation frequency increases with increasing pressure ratio. The tip-streaming mode is considered to be an extreme state of jetting mode, leading to higher formation frequency and smaller droplet size at low uniformity. Droplets are produced under tip-streaming mode and dripping mode in different states. Tip-streaming mode provides high productivity, while dripping mode provides more uniform droplets. Furthermore, through deeper study in the future, we expected to achieve the use of a semi-3D flow-focusing device to produce PEGDA droplets with both high productivity and uniformity.

## Figures and Tables

**Table 1:** The mode transition in the droplet breakup process.

Surfactant Concentration	Mode Transition	Pressure Ratio of Transition
5 vol %	jetting to dripping	1.8
10 vol %	jetting to dripping	1.9
15 vol %	jetting to tip-streaming	2.03
20 vol %	jetting to tip-streaming	2.1
25 vol %	jetting to tip-streaming	2.2

## Supplementary Materials

The following are available online at http://www.mdpi.com/2072-666X/9/4/139/s1, Video S1: PEGDA droplet breakup processes at five different bulk concentration of surfactant(5, 10, 15, 20, 25 vol %); Video S2: ETPTA droplet generation under jetting mode and dripping mode for 5 vol %; Video S3: ETPTA droplet generation under jetting mode and dripping mode for 11 vol %; Video S4: tip-streaming mode.
